# RNA-guided single/double gene repressions in *Corynebacterium glutamicum* using an efficient CRISPR interference and its application to industrial strain

**DOI:** 10.1186/s12934-017-0843-1

**Published:** 2018-01-09

**Authors:** Jaehyun Park, Hyojung Shin, Sun-Mi Lee, Youngsoon Um, Han Min Woo

**Affiliations:** 1 Department of Food Science and Biotechnology, Sungkyunkwan University (SKKU), 2066 Seobu-ro, Jangan-gu, Suwon, 16419 Republic of Korea; 2 Clean Energy Research Center, Korea Institute of Science and Technology, Hwarang-ro 14-gil 5, Seongbuk-gu, Seoul, 02792 Republic of Korea; 3 Present Address: GyeongSangBukdo Government Public Institute of Health & Environment, 22, Gosugol-gil Geumho-eup, Yeongcheon-si, 38874 Republic of Korea

**Keywords:** Metabolic engineering, Synthetic biology, CRISPR interference, *Corynebacterium glutamicum*

## Abstract

**Background:**

The construction of microbial cell factories requires cost-effective and rapid strain development through metabolic engineering. Recently, RNA-guided CRISPR technologies have been developed for metabolic engineering of industrially-relevant host.

**Results:**

To demonstrate the application of the CRISPR interference (CRISPRi), we developed two-plasmid CRISPRi vectors and applied the CRISPRi in *Corynebacterium glutamicum* to repress single target genes and double target genes simultaneously. Four-different single genes (the *pyc*, *gltA*, *idsA*, and *glgC* genes) repressions were successfully performed using the CRISPRi vectors, resulting significant mRNA reductions of the targets compared to a control. Subsequently, the phenotypes for the target gene-repressed strains were analyzed, showing the expected cell growth behaviors with different carbon sources. In addition, double gene repression (the *idsA* and *glgC* genes in a different order) by the CRISPRi resulted in an independent gene repression to each target gene simultaneously. To demonstrate an industrial application of the CRISPRi, citrate synthase (CS)-targeting DM1919 (l-lysine producer) strains with a sgRNA-gltA-r showed reduced CS activity, resulting in the improvement of l-lysine yield by 1.39-fold than the parental DM1919 (a lysine producer).

**Conclusions:**

Single or double gene repression were successfully performed using the CRISPRi vectors and sequence specific sgRNAs. The CRISPRi can be applied for multiplex metabolic engineering to enhanced lysine production and it will promote the further rapid development of microbial cell factories of *C. glutamicum*.

**Electronic supplementary material:**

The online version of this article (10.1186/s12934-017-0843-1) contains supplementary material, which is available to authorized users.

## Background

Controlling gene expression using Clustered Regulatory Interspaced Short Palindromic Repeats (CRISPR) inference (CRISPRi) and CRISPR activation (CRISPRa) was first demonstrated to modulate gene expression in *Escherichia coli* [1]. Subsequently, native or modified CRISPRi of *E. coli* has been applied to other bacteria and a yeast by repressing multiple genes in cyanobacteria [2], repressing essential genes to study the essentialities in mycobacteria [3], and to analyze fitness effects of guide RNA libraries to identify chemical-genetic interactions in *Saccharomyces cerevisiae* [4]. Comprehensive genetic tool kits for genome editing and CRISPRi have been also developed for *Bacillus subtilis* [5]. In addition, metabolic engineering with CRISPRi resulted in cost-effective and rapid strain development by controlling polyhydroxyalkanoate (PHA) biosynthesis flux, PHA composition [6], and balancing gene expression of the heterologous mevalonate pathway in *E. coli* [7].

*Corynebacterium glutamicum* is a predominantly aerobic, non-pathogenic, biotin-auxotrophic, Gram-positive bacterium that is used industrially for the production of amino acids, in particular the flavor enhancer l-glutamate and the feed additive l-lysine [8]. A CRISPRi system for the metabolic engineering of *C. glutamicum* has been developed to repress single genes [9] for improvement of l-lysine and l-glutamate production, and the CRISPRi system also has been applied to regulate multiple genes for shikimic acid production [10].

**Fig. 1:**
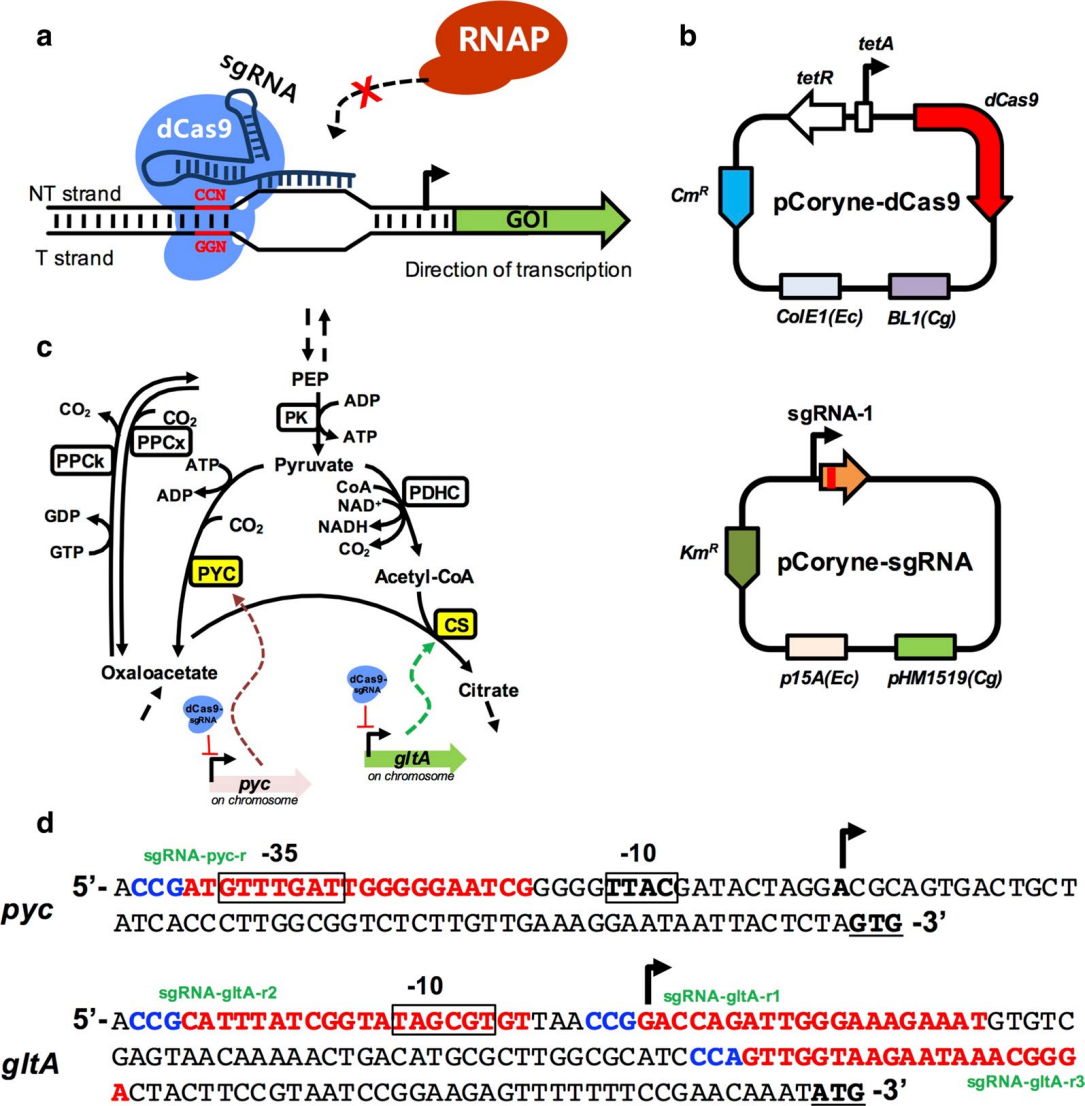
Application of the CRISPR interferences in *C. glutamicum*. **a** Scheme of the CRISPRi system that requires co-expressing a catalytically inactive version of Cas9 (dCas9) protein and a programmable single guide RNA (sgRNA) for the gene of interest (GOI). dCas9 recognizes the PAM sequence (5′-NGG-3′). A programmable sgRNA with dCas9 was designed to block the binding of RNA polymerase. **b** Two-plasmid system of the CRISPRi for *C. glutamicum*: pCoryne-dCas9 expresses dCas9 under the *tetA* promoter and pCoryne-sgRNA expresses a single sgRNA (base-paring region, dCas9 handle, and *S. pyogenes* terminator) under a constitutive promoter. **c** Application of the CRISPRi (dCas9-sgRNA complex) to either the wild-type or l-lysine producer (DM1919) by repressing mRNA expression of the chromosomal *pyc* gene or *gltA* gene. **d** Sequences of the PAM sites (blue) and protospacers (red) for CRISPRi of the *pyc* or *gltA* genes. The -35 and -10 regions in the promoter DNA sequence are shown in a box. Transcriptional start sites are shown with black arrows. The start codons for translation are underlined. Specific sgRNA names are shown next to the protospacer. The plasmids containing sgRNAs are listed in Table 1

In this study, we report the development of a two-plasmid CRISPRi and the detail studies with single guide RNAs for single or double repression of target genes in *C. glutamicum* wild-type (Fig. 1a). In addition, one of the known target gene was tested to demonstrate the CRISPRi application in a lysine producer for improving lysine yield, yielding a 1.39-fold increase to the parental strain. Thus, the CRISPRi vectors could be a cost-effective and time-saving metabolic engineering tool and will promote for researchers to investigate controlling gene expression for biochemical production in *C. glutamicum*.

## Results and discussion

### Construction of the CRISPR interference for *C. glutamicum*

**Fig. 2:**
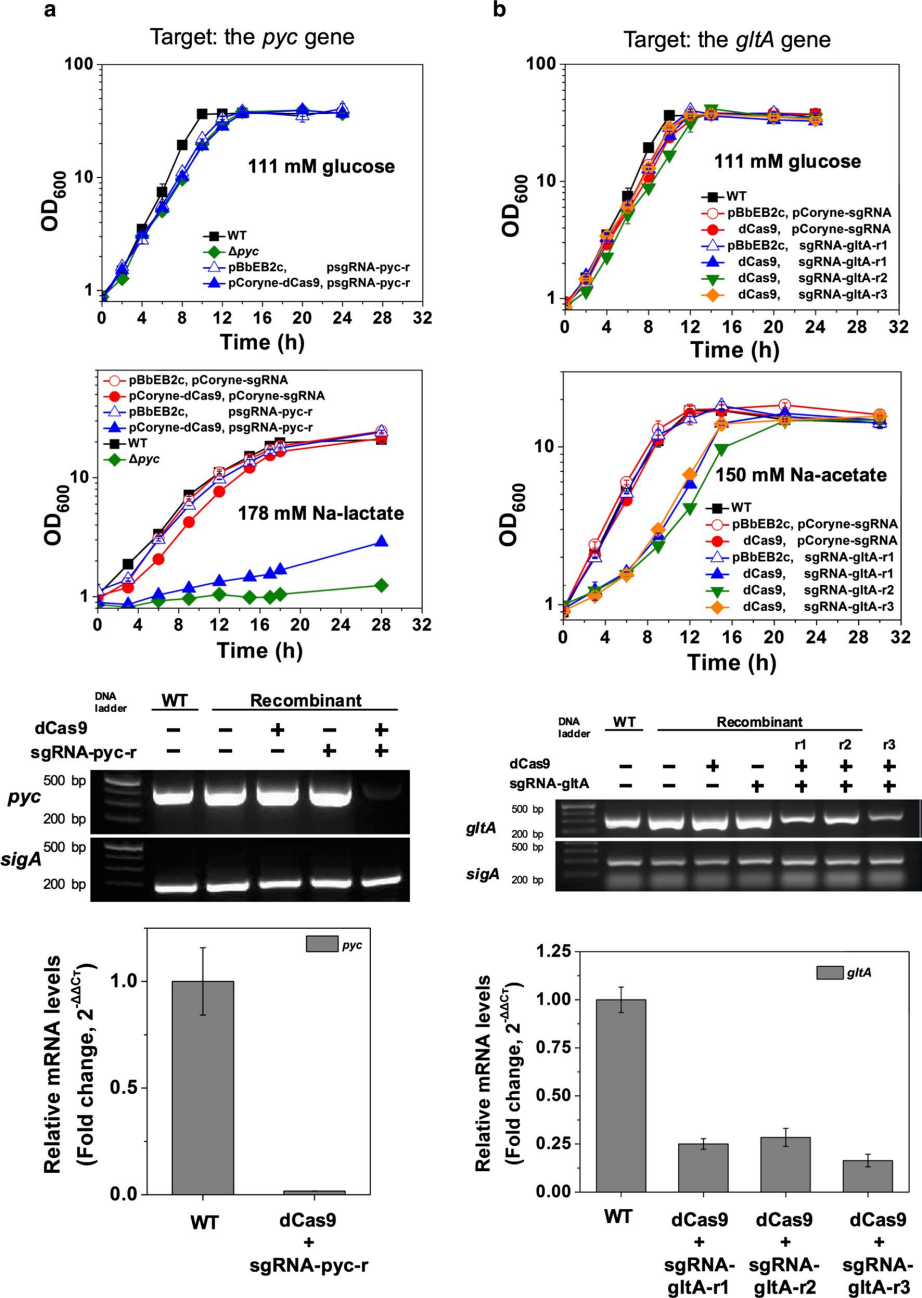
Single repression of the *pyc* gene and *gltA* gene by the two-plasmid CRISPRi system in *C. glutamicum* wild-type (WT). The wild-type and Pyc-targeting recombinant cells were cultivated with either 111 mM glucose or 178 mM sodium lactate as sole carbon (**a**; left panel). The wild-type and CS-targeting recombinant cells were cultivated with either 111 mM glucose or 150 mM sodium acetate as the sole carbon source (**b**; right panel). RT-PCR and quantitative RT-PCR were performed to investigate the levels of mRNA expression of the *pyc* gene, the *gltA* gene, and the *sigA* gene as a control for the wild-type and the recombinants with sgRNAs grown with 111 mM glucose. Data represents mean values of at least three cultivations and error bars represent standard deviations

To construct the two-plasmid system (pCoryne-dCas9 and pCoryne-sgRNA) of the CRISPRi (Table 1 and Fig. 1b), the CoryneBrick vector system [11] was used for expression of the *dCas9* gene encoding deactivated Cas9 nuclease (dCas9) under pTetA promoter, and a high-copy plasmid (pZ8-1) was modified to express a single guide RNA (sgRNA) under the constitutive promoter, based on CRISPRi of the Qi’ lab [12]. Compared to the previous CRISPRi [9], the *dCas9* gene was expressed under the control of pTetA promoter and there was no problem to obtain recombinant clones in this study. To check possible negative influences using our CRSPRi system, two basic strains were developed using pCoryne-dCas9 and pCoryne-sgRNA (empty vector) plasmids: Wild-type (Wt) pBbEB2c pCoryne-sgRNA and Wt pCoryne-dCas9 pCoryne-sgRNA. Both strains showed very slight growth inhibitions in CgXII minimal medium with 111 mM glucose, compared with the wild-type (Fig. 2). This could be due to cellular burden by harboring two plasmids. However, no growth differences were found between the two strains. Thus, we concluded that expressing dCas9 through the CRSPRi did not cause severe cellular defects in *C. glutamicum*.

**Table 1:** Bacteria strains and plasmids used in this study

Strain or plasmid	Relevant characteristics	References
Strains		
*E. coli* DH5α	F^−^(80d *lac*Z M15) (*lac*ZYA-*arg*F) U169 *hsd*R17(r^−^ m^+^) *rec*A1 *end*A1 *rel*A1 *deo*R96	[26]
*C. glutamicum* ATCC 13032	Wild-type strain, biotin auxotroph	ATCC
*C. glutamicum* Wt derivatives	Wild-type strain containing pCoryne-dCas9 and pCoryne-sgRNA-target_gene-r	This study
*C. glutamicum* DM1919	*pyc*(P458S), *hom*(V59A), 2 copies of *lysC*(T311I), Δ*pck,* l-lysine producer	Evonik Laboratory
Plasmids		
pBbEB2c	ColE1(Ec), pBL1(Cg), Cm^r^, P_*trc*_, BglBrick sites, CoryneBrick vector, TetR-*P*_*tetA*_	[11]
pdCas9-bacteria	p15A(Ec), Cm^r^, inactive bacterial Cas9 (*Streptococcus pyogenes*), addgene#44249	[1]
pCoryne-dCas9	pBbEB2c derivative containing the *dCas9* gene	This study
pZ8-1	p15A(Ec), pHM1519(Cg), Km^r^, *P*_*tac*_, *E. coli*–*C. glutamicum* shuttle vector	[27]
pgRNA-bacteria	ColE1 (Ec), Amp^r^, customizable guide RNA (gRNA), addgene#44251	[1]
pCoryne-sgRNA	pZ8-1 derivative, *Eco*R1, *Bgl*II and *Bam*HI compatible	This study
psgRNA-pyc-r	pCoryne-sgRNA containing sgRNA-pyc-r targeting transcriptional repression of the *pyc* gene	This study
psgRNA-gltA-r1	pCoryne-sgRNA containing sgRNA-gltA-r1 targeting transcriptional repression of the *gltA* gene	This study
psgRNA-gltA-r2	pCoryne-sgRNA containing sgRNA-gltA-r2 targeting transcriptional repression of the *gltA* gene	This study
psgRNA-gltA-r3	pCoryne-sgRNA containing sgRNA-gltA-r3 targeting transcriptional repression of the *gltA* gene	This study
psgRNA-idsA-r1	pCoryne-sgRNA containing sgRNA-idsA-r1 targeting transcriptional repression of the *idsA* gene	This study
psgRNA-idsA-r2	pCoryne-sgRNA containing sgRNA-idsA-r2 targeting transcriptional repression of the *idsA* gene	This study
psgRNA-idsA-r3	pCoryne-sgRNA containing sgRNA-idsA-r3 targeting transcriptional repression of the *idsA* gene	This study
psgRNA-glgC-r1	pCoryne-sgRNA containing sgRNA-glgC-r1 targeting transcriptional repression of the *glgC* gen	This study
psgRNA-glgC-r2	pCoryne-sgRNA containing sgRNA-glgC-r2 targeting transcriptional repression of the *glgC* gene	This study
psgRNA-glgC-r2-idsA-r1	pCoryne-sgRNA containing sgRNA-glgC-r2, sgRNA-idsA-r1 targeting transcriptional repression of the *glgC* and *idsA* gene simultaneously	This study
psgRNA-idsA-r1-glgC-r2	pCoryne-sgRNA containing sgRNA-idsA-r1, sgRNA-glgC-r2 targeting transcriptional repression of the *idsA* and *glgC* gene simultaneously	This study

### Application of the CRISPR interference to repress single gene in *C. glutamicum*

First, a Pyc-targeting recombinant (Wt pCoryne-dCas9 psgRNA-pyc-r) was constructed to investigate the performance of the CRISPRi. The 20 bp protospacer of psgRNA-pyc-r was designed as nontemplate (NT) strand of the promoter region to block the transcription (Fig. 1d) because the efficiency of transcriptional repression using NT strand targeting sgRNAs showed much higher than using template strand targeting sgRNAs [1, 9]. The possible off-target sites using the sgRNA-pyc-r were examined using the Cas-OFFinder [13], resulting no off-target sites found. Then, the Pyc-targeting strain (Wt pCoryne-dCas9 psgRNA-pyr-r) was cultivated in CgXII minimal medium either with 111 mM glucose or 178 mM sodium lactate, resulting in the similar growth as the control (Wt pCoryne-dCas9 pCoryne-sgRNA) on glucose but strong growth inhibition on lactate (Fig. 2a). This phenotype has been consistently shown in the *pyc* deletion mutant [14]. Subsequently, the mRNA levels of the *pyc* gene were analyzed by both RT-PCR and RT-qPCR for cells grown on glucose. No cDNA product was shown in the gel for the Pyc-targeting strain alone. The mRNA level of the *pyc* gene was significantly down-regulated (1.69% of native mRNA expression) in the Pyc-targeting recombinant when glucose was used as the sole carbon source. Thus, the dCas9/sgRNA-pyc-r complex indeed repressed mRNA expression of the *pyc* gene using the CRSPRi when lactate or glucose were used.

In parallel, the *gltA* gene encoding for citrate synthase (CS) was selected to increase lysine production using the CRISPRi in *C. glutamicum* because of the importance of CS activity in central metabolism and its influence on l-lysine synthesis [15] and the gene essentiality. The wild-type and DM1919 (a l-lysine producer) strains were used for the single gene repression and its applications. Three different sgRNAs targeting different protospacers on the nontemplate DNA strand of the promoter region of the *gltA* gene were designed (Fig. 1c, d; Table 1). After confirming no off-target sites, each sgRNA was expressed in the CRISPRi, yielding three different recombinants. The CS-targeting recombinants (with sgRNA-gltA-r1, -r2, and -r3), wild-type strains expressing either dCas9 or sgRNA-gltA-r1 (non-CS targeting recombinants), and a wild-type strain harboring the two empty plasmids (pBbEB2c; pCoryne-sgRNA) as a control were cultivated on 111 mM glucose minimal CgXII medium. As a result, CS- or non-CS-targeting recombinant strains showed similar growth to the wild-type. Thus, the CRISPRi either with or without the target sgRNA did not cause growth inhibition on glucose minimal medium, which was consistent with the result of the Pyc-targeting strain (Fig. 2b). Subsequently, we analyzed *gltA* gene mRNA expression in the CS targeting recombinants and non-CS targeting recombinants using RT-PCR and qRT-PCR when glucose was used as the sole carbon source. The mRNA expression level of the *gltA* gene in non-CS targeting recombinant strains and the control were similar to the levels in the wild-type. However, the mRNA levels of CS-targeting recombinants (sgRNA-gltA-r1, -r2, and -r3) were decreased to 25, 28.5, and 16.4% of the levels of the wild-type, respectively, although there was no growth inhibition.

In addition, the wild-type and CS or non-CS targeting recombinants were cultivated on CgXII medium containing 150 mM sodium acetate as the sole carbon source. The three CS-targeting recombinants showed slower growth rates than the wild-type. This growth inhibition could be due to the repressed *gltA* mRNA expression because 76% of acetyl-coA from acetate is converted to citrate and isocitrate by CS and aconitase [16]. Interestingly, the residual mRNA expression of CS was still enough for the CS-targeting recombinants to grow to the same OD_600_ of the wild-type.

### Application of the CRISPR interference to repress double genes simultaneously in *C. glutamicum*

**Fig. 3:**
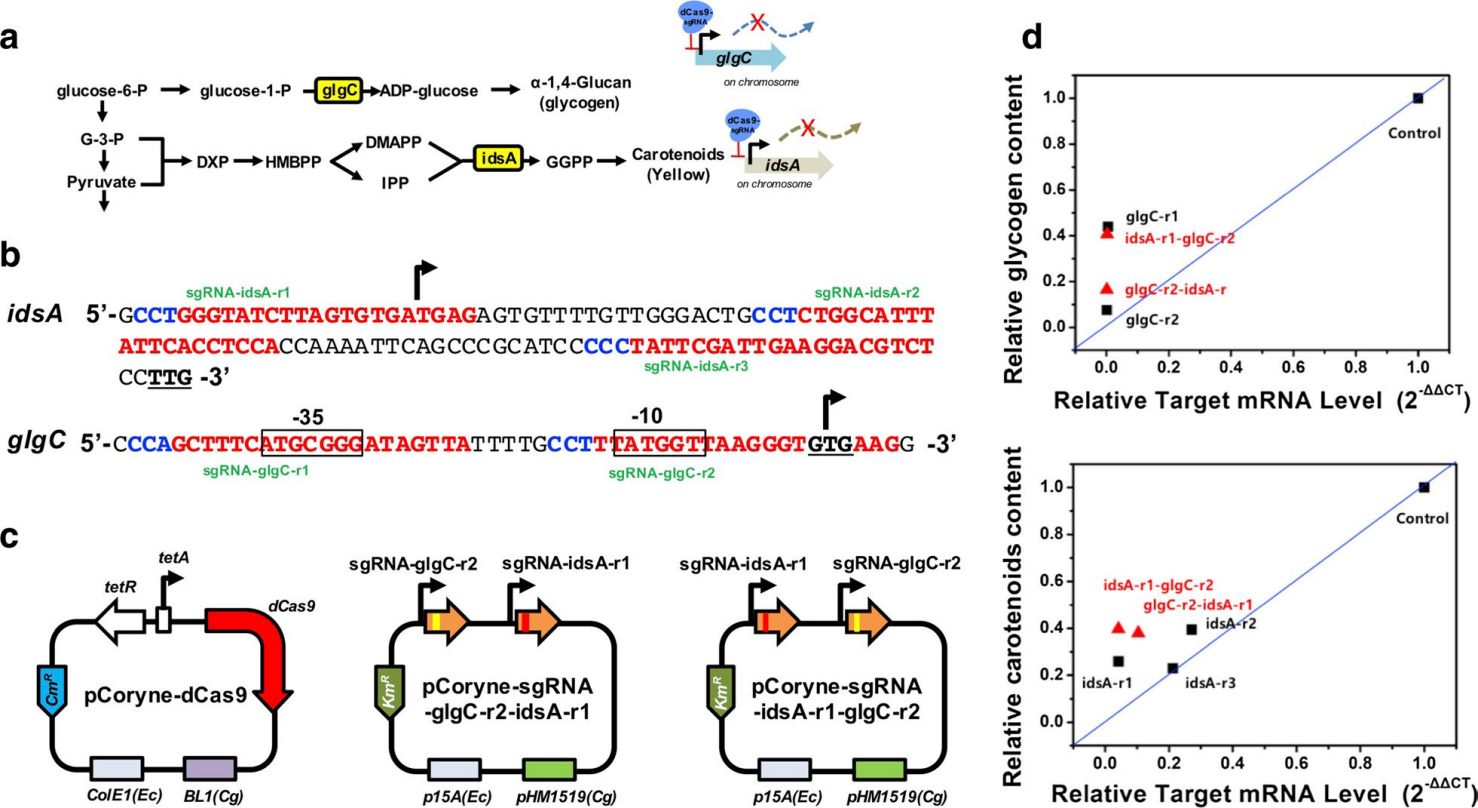
Double repression of the *idsA* gene and *glgC* gene by the two-plasmid CRISPRi system in *C. glutamicum* wild-type (WT). **a** The biosynthesis pathways of glycogen and carotenoids were described. **b** Sequences of the PAM sites (blue) and protospacers (red) for CRISPRi of the *idsA* or *glgC* genes. The -35 and -10 regions in the promoter DNA sequence are shown in a box. Transcriptional start sites are shown with black arrows. The start codons for translation are underlined. Specific sgRNA names are shown next to the protospacer. **c** Two-plasmid system of the CRISPRi for *C. glutamicum* for double gene repressions of the *glgC* and *idsA* gene. **d** The relative mRNA levels of the *glgC* gene were correlated with relative glycogen contents (mg intracellular glycogen [glucose] per g dry cell weight) to a control (wild-type strain expressing dCas9 and null sgRNA). The relative mRNA levels of the *idsA* gene were correlated with relative carotenoid contents to a control (wild-type strain expressing dCas9 and null sgRNA). Black square showed single gene repression experiments and red triangle showed double gene repression experiments. See the details in Table 1

To investigate simultaneous double gene repression using the CRISPRi in *C. glutamicum*, the *glgC* and *idsA* genes were selected because there were no reports that the target genes are co-regulated each other. ADP-glucose pyrophosphorylase encoded by the *glgC* gene is a key enzyme in glycogen synthesis and a major geranylgeranyl pyrophosphate (GGPP) synthase encoded by the *idsA* gene is a major enzyme in the carotenoid biosynthesis pathway (Fig. 3a). Thus, the phenotype of the *glgC* gene deletion mutant or the *idsA* gene deletion mutant were have been studied by measuring intracellular glycogen contents [17] or carotenoid contents [18], respectively.

**Fig. 4:**
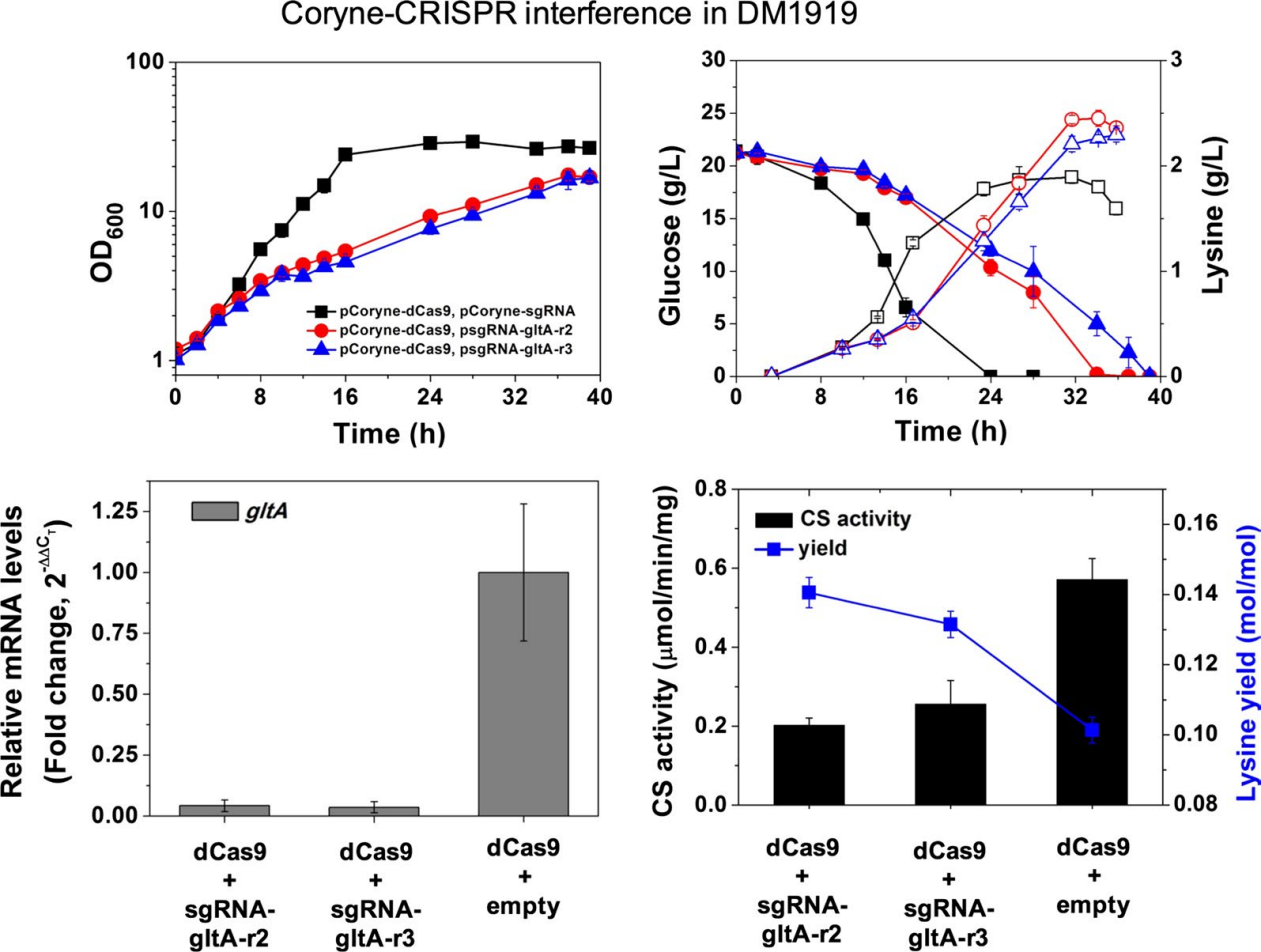
Application of the CRISPRi system for enhancement of lysine production in DM1919, the l-lysine producer. Two different sgRNAs (gltA-r2 and gltA-r3) were expressed in DM1919 strain to repress *gltA* gene expression (note the details in Table 1 plasmids). The cell growth (closed symbol), glucose consumption (closed symbol), and l-lysine production (open symbol) were measured for the DM1919 strains (upper panel). Levels of mRNA expression of the *gltA* gene and the *sigA* gene as a control, and citrate synthase (CS) activity (μmol/min/mg of total protein) in the DM1919 strains were measured (lower panel). One unit (U) is defined as the amount of enzyme that converts 1 μmol of acetyl-coA to citrate per a minute at 30 °C. The l-lysine yields (mol/mol) were calculated (mole of l-lysine produced per mole glucose consumed). Data represents mean values of at least three cultivations and error bars represent standard deviations

In prior to double repression, single gene repression for the *glgC* gene or the *idsA* target gene must be studied. Two different sgRNAs for the *glgC* gene repression and three different sgRNAs for the *idsA* gene repression were designed as a NT strand targeting sgRNA and cloned into pCoryne-sgRNA vectors (Fig. 3b, c). As expected from the gene deletion studies [17, 18], GlgC-targeting strains showed reduced mRNA expressions (less than 0.01% expression of the native mRNA) and intracellular glycogen contents, when cells were harvested at 6 h, were reduced by 57 and 92% compared to a control (the wild-type strain expressing dCas9 and null sgRNA) (Fig. 3d and Additional file 1: Figure S3). In parallel, IdsA-targeting strains showed also reduced mRNA expressions (4, 27, 21% of mRNA expression of the native expression) with sgRNA-idsA-r1, sgRNA-idsA-r2, and sgRNA-idsA-r3, respectively. The relative carotenoid contents in IdsA-targeting strains were positively correlated with their mRNA expressions except the sgRNA-idsA-r3 (Fig. 3d and Additional file 1: Figure S3).

Subsequently, sgRNA-glgC-r2 and sgRNA-idsA-r1, which showed the best gene repression activities, were cloned into a pCoryne-sgRNA for double gene repression and the order of sgRNAs was differently determined as: glgC-r2-idsA-r1 and idsA-r1-glgC-r2 (Fig. 3c). Both GlgC- and IdsA-targeting strains were analyzed by measuring mRNA level and its phenotype. As a result, features in a genetic or phenotypic aspect were observed for double gene repression using the two-plasmid CRISPRi vectors (Fig. 3d).

Each sgRNA expression is designed to transcribe by its own consecutive promoter and terminator. Thus, each gene repression using the CRISPRi has been theoretically orthogonal for multiple gene repressions in *E. coli* [1, 6]. However, the order of sgRNA for double gene repression slightly matters in *C. glutamicum*, depending on the context of the target gene. The mRNA levels of the *idsA* gene were not changed only when the sgRNA was positioned in front of anther sgRNA for double repression, compared to single repression of the *idsA* gene. In this case, the first sgRNA expression may serve tighter gene repression than the second sgRNA expression in a pCoryne-sgRNA plasmid. However, the mRNA levels of the *glgC* gene were less influenced by the sgRNA order due to the leaderless promoter for gene expression. Interestingly, the carotenoid contents were not changed by the order although the mRNA levels were slightly altered by the order in case of the *idsA* gene repression. However, the glycogen contents were significantly different by the order. Thus, it could be necessary to map the correlations between target gene expression and its phenotype, which the CRISPRi with multiple targets may be useful in a high-throughput manner.

Overall, the double gene repression worked successfully in order to reduce target mRNA expressions, resulting corresponding altered phenotypes in *C. glutamicum*. Still, fine-tuning of gene expression for dCas9 and sgRNAs in the two-plasmid CRISPRi vectors must be required in order to perform tunable gene repressions for multiple gene targets.

### Application of the CRISPR interference to improve lysine production in a lysine producer, DM1919

Based on the results of the reduced mRNA expression of the *gltA* gene by the CRISPRi, the *gltA* gene in the DM1919 strain was targeted to investigate whether l-lysine yield could be improved. For cell growth, CS-targeting recombinants of DM1919 showed growth inhibition and lower glucose consumption rates compared to the DM1919 strain expressing dCas9 alone (Fig. 3), although there was no growth inhibition shown in CS-targeting recombinants from Wt. Growth inhibition was also observed with the lysine producer JVO2 strains (*pyc*(P458S) *lys*C(T311I) Δ*prp*C1 Δ*prp*C2) with an engineered *gltA* promoter [15], in which the strains showed 20–30% CS activity of the native CS activity. Subsequently, the mRNA levels of the *gltA* genes in CS-targeting DM1919 strains were analyzed (Fig. 3). The mRNA levels of the *gltA* gene were significantly down-regulated (4.2 and 3.6% of native mRNA expression) in the CS-targeting DM1919 strains with sgRNA-gltA-r2 and -r3, respectively. The strong mRNA reduction of the *gltA* gene could affect the growth inhibitions of the CS-targeting DM1919 compared to DM1919 alone. However, the reason why the mRNA repressions of the *gltA* gene were stronger in DM1919 than in the wild-type was unclear even though the same sgRNAs were used. For l-lysine production, CS-targeting DM1919 strains with either sgRNA-gltA-r2 or -r3, exhibiting 35.5 or 44.8% native CS activity (Fig. 3), respectively, showed a 1.39-fold or 1.30-fold higher l-lysine yield, respectively, compared to non-CS-targeting DM1919 expressing dCas9 only. Similarly, the JVO2-A23 with 23% of native CS activity showed a 1.47-fold increase of l-lysine yield when compared to JVO1 with 100% native CS activity [15].

The CRISPRi system can expand the repression of more than double gene in *C. glutamicum* by inserting genes expressing multiple sgRNAs, which could be explored to construct microbial cell factories [10]. In addition, the fine-tuning of gene repression for multiple targets must be further developed for *C. glutamicum* with different protospacer lengths, functional protospacer adjacent motifs (PAMs), mutated seed sequences in the protospacer, exchanging the promoter strength of sgRNA transcript, or controlling dCas9 gene expression. Moreover, genome-wide applications for the metabolic engineering of *C. glutamicum* could be also useful to modulate gene expression using CRISPR interference combined with sgRNA libraries. Besides CRISPRi, CRISPRa could be interesting for RNA-guided gene activation in *C. glutamicum* by utilizing dCas9 fused to a subunit that recruits more RNA polymerase.

## Conclusions

In summary, we report the development and application of the CRISPRi capable of repressing single or double target genes including an essential gene (*pyc*, *gltA*, *idsA*, and *glgC*) in *C. glutamicum* by choosing sequence-specific protospacers. For an application to the industrial host strain through interfering mRNA transcription with dCas9 and different sgRNAs for the *gltA* gene, the CS-targeting recombinants exhibited various CS activities and indeed improved the level of l-lysine yield. The CRISPRi vectors employed in this study will be useful to promote cost-effective for providing high-throughput host engineering of *C. glutamicum* for constructing microbial cell factories.

## Methods

### Bacterial strains and culture conditions

All bacterial strains used or constructed in this work are listed in Table 1. *E. coli* strains were grown in LB medium (containing per liter: 10 g tryptone, 5 g yeast extract, and 5 g NaCl) at 37 °C and 200 rpm. For lysine production, *C. glutamicum* ATCC 13032 and its derivatives were cultivated in BHIS medium (containing per liter: 37 g brain heart infusion, 91 g sorbitol) [19] at 30 °C and 120 rpm overnight and then incubated aerobically in CgXII defined medium (50 mL in 250 mL baffled Erlenmeyer flasks) [19] containing either 111 mM glucose, 150 mM sodium acetate, or 178 mM sodium lactate supplemented with 25 μg/mL kanamycin and 7.5 μg/mL chloramphenicol at 30 °C on a rotary shaker at 200 rpm. For gene identification study, *C. glutamicum* strains were pre-cultivated in 50 mL CgXII medium containing 2% (w/v) glucose supplemented with 25 μg/mL kanamycin and 7.5 μg/mL chloramphenicol at 30 °C on a rotary shaker at 120 rpm [20]. Once OD_600_ reached 12, the culture was harvested and the cell pellet was washed with CgXII medium without carbon source and transferred to 50 mL CgXII medium containing 270 mM MeOAc in 125 mL serum bottle with a screw cap at 30 °C on a rotary shaker at 120 rpm. All chemicals used in this study were purchased from Sigma-Aldrich (St. Louis, MO). For induction, 100 nM anhydrotetracycline (aTC) was added.

### Construction of CRISPRi plasmids and recombinant *C. glutamicum* strain

*Eco*RI/*Xho*I-digested DNA fragments containing the *dCas9* gene from the pdCas9-bacteria plasmid [12] were inserted at the *Eco*RI and *Xho*I sites of the CoryneBrick plasmid pBbEB2c [11], yielding pCoryne-dCas9. In parallel, pZ8-1 was modified to construct pCoryne-sgRNA by removing the promoter region using a pair of primers (pCoryne-sgRNA-fw/rv). Based on the cloning protocol of CRISPRi [12], the DNA fragments containing a constitutive promoter, a sgRNA (target-specific protospacer region, *dCas9* handle, and *S. pyogenes* terminator), a transcriptional terminator (*rrnB*) was amplified from the DNA template of the pgRNA-bacteria plasmid [1] using target-specific sgRNA primers (Additional file 1: Table S1). Then, a DNA cassette containing the target-specific sgRNA was inserted to pCoryne-sgRNA, yielding a psgRNA-target plasmid. The pCoryne-dCas9 and pCoryne-sgRNA plasmids served as standard CRISPRi plasmids for the sequence-specific control of gene expression in *C. glutamicum*. The plasmids used or constructed in this work are listed in Table 1 and the primers used in this study are listed in Additional file 1: Table S1. The resulting plasmids were introduced into *C. glutamicum* by electroporation, and strain validation was performed by colony PCR [19]. The resulting strains are listed in Table 1.

### Metabolite measurements using HPLC

Glucose and l-lysine in the supernatant were quantified by HPLC as described previously [11, 21]. The details were described in Additional file 1. Briefly, culture supernatant was passed through a syringe filter (pore size of 0.2 μm) after centrifugation at 10,000×*g* for 10 min. The glucose concentration was determined by high-performance liquid chromatography (HPLC system Agilent 1260, Waldbronn, Germany) equipped with a refractive index detector (RID), UV detector, and an Aminex HPX-87 H Ion Exclusion Column (300 mm by 7.8 mm, Bio-Rad, Hercules, CA) under the following conditions: sample volume of 20 μL, mobile phase of 5 mM H_2_SO_4_, flow rate of 0.6 mL/min, and column temperature of 65 °C. For the quantification of l-lysine, the sample was analyzed by reversed-phase high-pressure liquid chromatography after pre-column derivatization with a fresh mixture of o-phthaldialdehyde and β-mercaptoethanol according to a previous study using an Agilent 1100 LC system (Agilent Technologies, Santa Clara, CA) equipped with a ZORBAX Eclipse XDB column (150 × 4.6 mm) and a diode array detector (338, 262 nm). Followings the user’s manual, substances were eluted with a flow rate of 1 mL/min for the following 30 min at 40 °C with a gradient of mobile phase A (10 mM Na_2_HPO_4_, 10 mM Na_2_B_4_O_7_ in H_2_O; pH 8.2) containing 8 mg/L NaN_3_ and mobile phase B (acetonitrile:methanol:H_2_O = 45:45:10) (volumetric %).

### RT-PCR and RT-qPCR analysis

Total RNA purification and reverse transcription (RT)-PCR analysis were performed as described previously by our group [22]. For quantitative RT-PCR (qRT-PCR), with total RNA, reverse transcription was performed using SuperScript II RTase (Invitrogen, USA) according to the manufacturer’s instructions. The cDNAs were amplified using the designated primers (Additional file 1: Table S2). qRT-PCR was performed on the QuantStudio 3 Real-Time PCR System (Applied Biosystems, USA) using SYBR Green PCR Kit (Applied Biosystems, USA) or TagMan Gene expression master mix (Applied Biosystems, CA, USA), according to the manufacturer’s instructions. Thermal cycling conditions were 95 °C for 10 min followed by 40 cycles of 95 °C for 15 and 30 s at optimal Tm (60 °C). The data were analyzed using the QuantStudio Design & Analysis software (Applied Biosystems, USA). The primers used for qRT-PCR are listed in Additional file 1: Table S2. The expression levels of each mRNA were normalized to a control (the *sigA* gene; housekeeping gene) [23] and were calculated using the 2^−ΔΔCt^ method [24].

### Analysis of total carotenoids

The extraction method has been followed from previous study [18]. Briefly, the pigments were extracted from the cell pellets with methanol/acetone (7:3) at 60 °C for 30 min with vigorous vortexing every 10 min. The extract mixture was centrifuged at 13,000×*g* for 5 min. The supernatants were used for determination of the total carotenoid contents by measuring absorbance at 470 nm using Eppendorf BioSepectrometer^®^ kinetic (Eppendorf AG, Hamburg, Germany).

### Measurement of intracellular glycogen content

The glycogen contents of *C. glutamicum* were determined by the previous enzymatic method [17]. Briefly, a 5 mL-culture sample were centrifuged, and the cells were washed with a Tris-based buffer (pH 6.3). The cell pellets were re-suspended in 1 mL of 40 mM potassium acetate buffer (pH 4.2). After a glass-bead beating, the supernatant was collected. Each sample was divided into two 100 µL aliquots (sample A and B). 2 µL of amyloglucosidase (10 mg/mL; Roche Diagnostics, Mannheim, Germany) was added to only sample A to degrade glycogen to free glucose, whereas sample B served as a reference. Both A and B samples were incubated for 2 h at 57 °C with shaking at 850 rpm. Subsequently, the glucose concentration in the two samples was determined using a coupled enzymatic assay with hexokinase and glucose 6-phosphate dehydrogenase (Sigma-Aldrich, St. Louis, MO) by measuring the NADH formed at 340 nm. Finally, the glycogen content was calculated in mg per g of cells (dry weight) after subtraction of the glucose concentration of the reference sample B from that of the test sample A.

### Measurement of citrate synthase activity

The quantification of citrate activity was performed as described previously [25], except that cells were disrupted by a glass bead-beater. Briefly, citrate synthase was assayed spectrophotometrically at 412 nm and 30 °C by measuring the appearance of free CoA coupled with 5,5′-dithiobis-(2-nitro-benzoate) (DTNB; ε = 13,600/M/cm at 412 nm). One unit (U, μmol/min) is defined as the amount of enzyme that converts 1 μmol of acetyl-coA to citrate for a minute at 30 °C.

## Additional file

**Additional file 1: Table S1.** Oligonucleotides used for gene cloning in this study. **Table S2.** Oligonucleotides used for RT-PCR and qRT-PCR in this study. **Figure S1.** Gel images of Fig. 2. **Figure S2.** Gel images of Fig. 4. **Figure S3.** Relative mRNA expression and phenotype analysis of single gene repressions in *C. glutamicum* strains for the double gene repression study. **Figure S4.** Relative mRNA expression and phenotype analysis of double gene repressions in *C. glutamicum* strains.

## Abbreviations

CRISPR: Clustered Regulatory Interspaced Short Palindromic Repeats
sgRNA: single guided RNA
PAM: protospacer adjacent motifs
NGS: next-generation sequencing
aTC: anhydrotetracycline
