# Supplementary material for: RNA-guided single/double gene repressions in Corynebacterium glutamicum using an efficient CRISPR interference and its application to industrial strain
Source: Microb Cell Fact. 2018 Jan 9;17:4. doi: 10.1186/s12934-017-0843-1 (PMC5759794; doi:10.1186/s12934-017-0843-1)
Supplement: Supplementary file 1 — Additional file 1: Table S1. Oligonucleotides used for gene cloning in this study. Table S2. Oligonucleotides used for RT-PCR and qRT-PCR in this study. Figure S1. Gel images of Fig. 2. Figure S2. Gel images of Fig. 4. Figure S3. Relative mRNA expression and phenotype analysis of single gene repressions in C. glutamicum strains for the double gene repression study. Figure S4. Relative mRNA expression and phenotype analysis of double gene repressions in C. glutamicum strains. [file 12934_2017_843_MOESM1_ESM.docx]

**RNA-guided single/double gene repressions in *Corynebacterium glutamicum* using an efficient CRISPR interference and its application to industrial strain**

Jaehyun Park^1,a^ Email: 7131wo@gmail.com

Hyojung Shin^2,a,b^ Email: hyojung336@gmail.com

Sun-Mi Lee^2^ Email: smlee@kist.re.kr

Youngsoon Um^2^ Email: yum@kist.re.kr

Han Min Woo^1^* Email: hmwoo@skku.edu

^1^Department of Food Science and Biotechnology, Sungkyunkwan University (SKKU), 2066 Seobu-ro, Jangan-gu, Suwon 16419, Republic of Korea

^2^Clean Energy Research Center, Korea Institute of Science and Technology, Hwarang-ro 14-gil 5, Seongbuk-gu, Seoul 02792, Republic of Korea

^a^JP and HS contributed equally to this work.

^b^Present address: GyeongSangBukdo Government Public Institute of Health & Environment

*Corresponding author at: Department of Food Science and Biotechnology, Sungkyunkwan University (SKKU), 2066 Seobu-ro, Jangan-gu, Suwon 16419, Republic of Korea, E-mail Tel.: +82 31 290 7808; E-mail address: hmwoo@skku.edu (H.M. Woo).

**Supplementary Table S1. Oligonucleotides used for gene cloning in this study.**

| Name | Relevant characteristics (5’ 🡪 3’) | Source |
| --- | --- | --- |
| pCoryne-sgRNA-*XhoI*-fwd | ATActcgagCAAGACGTTTCCCGTTGAA | This study |
| pCoryne-sgRNA-*EcoRI*-rev | GGgaattcTCAGGCAACTATGGATGAACGAAATAGA | This study |
| sgRNA*-SpeI*-rev | ATactagtATTATACCTAGGACTGAGCTAGC | This study |
| sgRNA*-pyc*-*SpeI*-fwd | TAATactagtCGATTCCCCCAATCAAACATGTTTTAGAGCTAGAAATAGCAA | This study |
| sgRNA*-gltA*-r1-*SpeI*-fwd | TAATactaagtACACGCTATACCGATAAATGGTTTTAGAGCTAGAAATAGCAA | This study |
| sgRNA*-gltA*-r2-*SpeI*-fwd | TATactagtATTTCTTTCCCAATCTGGTCGTTTTAGAGCTAGAAATAGCAA | This study |
| sgRNA*-gltA*-r3-*SpeI*-fwd | TATactagtTCCCGTTTATTCTTACCAACGTTTTAGAGCTAGAAATAGCAA | This study |
| sgRNA*-idsA*-r1-*SpeI*-fwd | TATactagtCTCATCACACTAAGATACCCGTTTTAGAGCTAGAAATAGCAA | This study |
| sgRNA*-idsA*-r2-*SpeI*-fwd | TATactagtTGGAGGTGAATAAATGCCAGGTTTTAGAGCTAGAAATAGCAA | This study |
| sgRNA*-idsA*-r3-*SpeI*-fwd | TATactagtAGACGTCCTTCAATCGAATAGTTTTAGAGCTAGAAATAGCAA | This study |
| sgRNA*-glgC*-r1-*SpeI*-fwd | TATactagtTAACTATCCCGCATGAAAGCGTTTTAGAGCTAGAAATAGCAA | This study |
| sgRNA*-glgC*-r2-*SpeI*-fwd | TATactagtCTTCACACCCTTAACCATAAGTTTTAGAGCTAGAAATAGCAA | This study |

Note: The restriction enzyme sites were shown as lower cases. Target specific-protospacer regions of the sgRNAs were underlined.

**Supplementary Table S2. Oligonucleotides used for RT-PCR and qRT-PCR in this study.**

| Name | Relevant characteristics (5’ 🡪 3’) | Source |
| --- | --- | --- |
| RT-*gltA*-fwd | CCAATGGCAACCTTGGCTTC | This study |
| RT- *gltA*-rev | ACAAACATGTTGGCCTGTGC | This study |
| RT-*pyc*-fwd | TAAAAGCGCTGAAGGCCAGA | This study |
| RT-*pyc*-rev | TCTTCAGTCACGGTGTGCTC | This study |
| qRT-gltA-fwd | CGCTGATCTGGCTGAGAATG | This study |
| qRT-gltA-rev | GGTTGGTAGCTCACCGTTGA | This study |
| qRT-pyc-fwd | GGAATTCTTGGTCGATGAAA | This study |
| qRT-pyc-rev | GTGTGCTCAACCTGGATACG | This study |
| RT-*sigA*-fwd | TTTGGGATGAAGACGAATCC | This study |
| RT-*sigA*-rev | TTCTTCCATTTCCTCCATGC | This study |
| qRT_sigA_fwd | GCAGCATCGATGGAACCTCA | This study |
| qRT_sigA_rev | GCCTTGCGAGCTGTCTTCTT | This study |
| qRT-sigA-Probe* | CCTGCGGCCAAGGCTCCCGC | This study |
| qRT-glgC-fwd | GTGGAACGTGTCTGGTCCAA | This study |
| qRT-glgC-rev | GGAACCAGTGAACCATCGCT | This study |
| qRT-glgC-Probe* | TCCTGCGCAGCAGCGCCTGG | This study |
| qRT-idsA-fwd | TGGCCCATCTTCGCAGTTTC | This study |
| qRT-idsA-rev | GCGTCAAGGACGGACTCAAG | This study |
| qRT-idsA-Probe* | TTCCTGGCGGCGCAAGGCCA | This study |

Note: *Oligo was modified with 5’-6-FAM and 3’-TAMRA as a dual-labeled DNA probe.

**Figure S1.** Gel images of the Figure 2. See the legend of the figure 2 in the text.

**
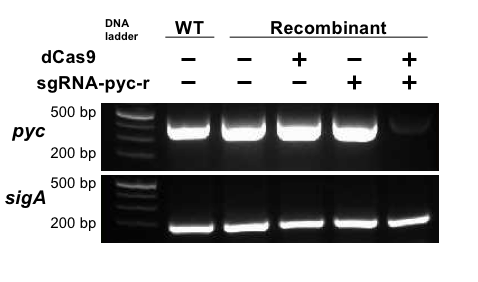

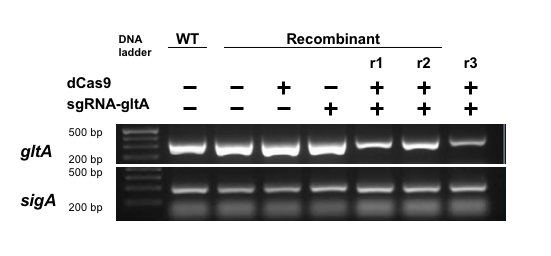
**

**Figure S2.** Gel images of the Figure 4. See the legend of the figure 4 in the text.

**
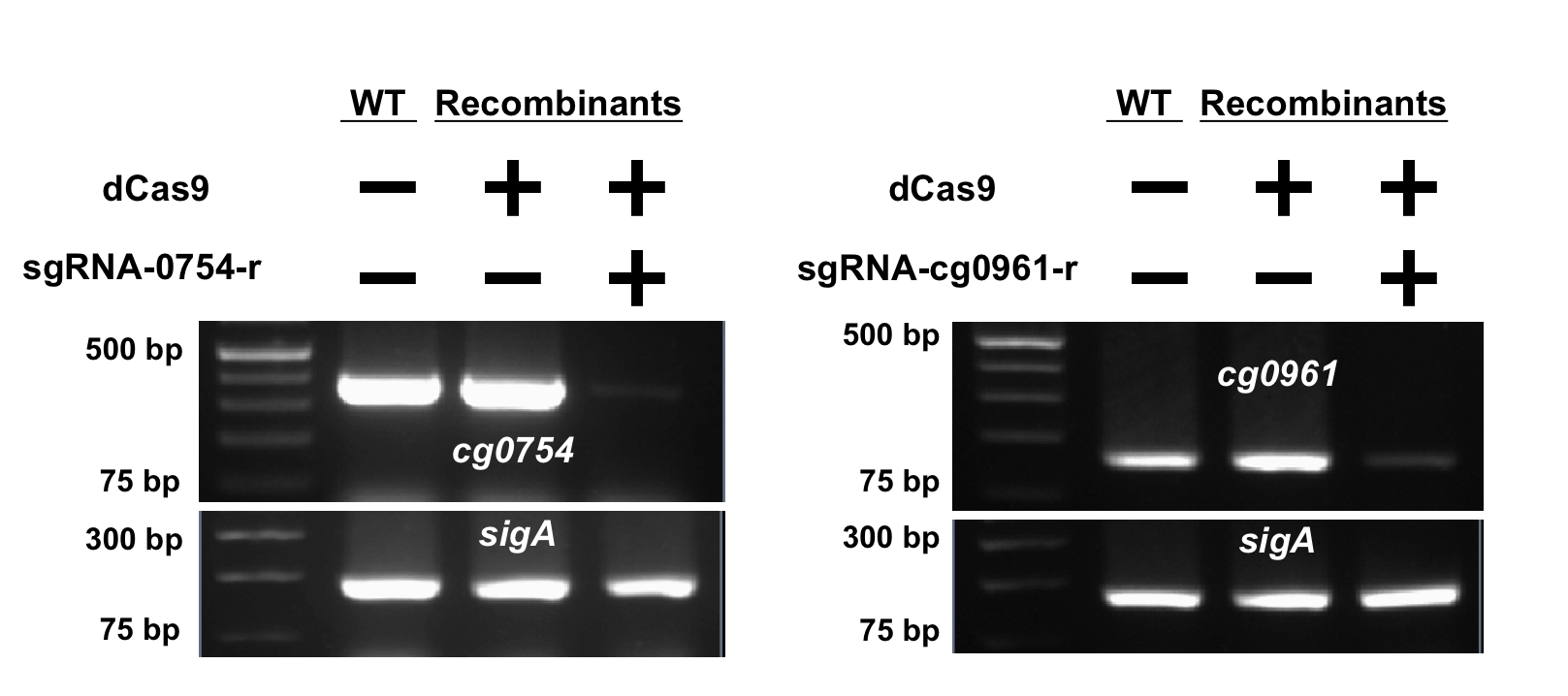
**

**Figure S3.** Relative mRNA expression of the target gene and phenotype analysis *C. glutamicum* strains with either the single *glgC* gene or *idsA* gene repression for the double gene repression study. Relative mRNA levels and glycogen content were measured in GlgC-targeting strains. On the other hand, relative mRNA levels and relative carotenoid contents were measured in IdsA-targeting strains. See the details in the Table 1 for strain characteristics.

**
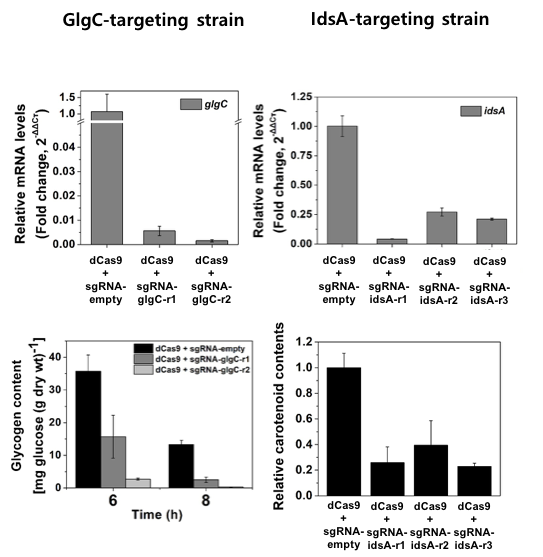
**

**Figure S4.** Relative mRNA expression of the target genes and phenotype analysis in *C. glutamicum* strains with both the *glgC* and *idsA* genes repression. Relative mRNA levels, glycogen content, and relative carotenoid contents were measured in the strains. See the details in the Table 1 for strain characteristics.

**
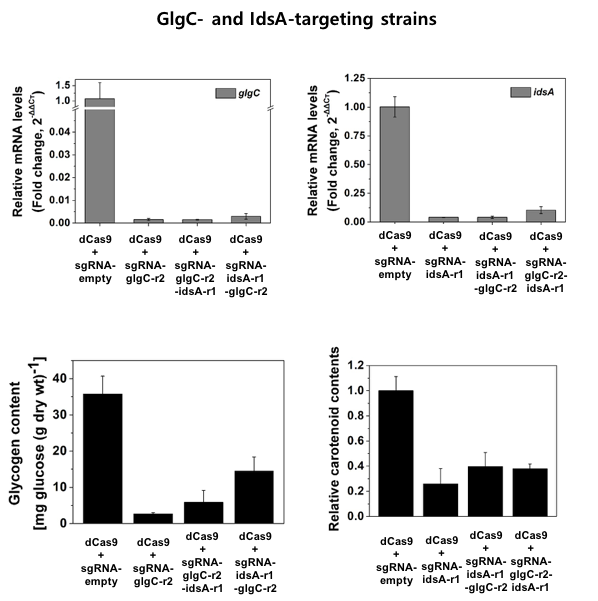
**
